# Soil Respiration in Different Agricultural and Natural Ecosystems in an Arid Region

**DOI:** 10.1371/journal.pone.0048011

**Published:** 2012-10-17

**Authors:** Liming Lai, Xuechun Zhao, Lianhe Jiang, Yongji Wang, Liangguo Luo, Yuanrun Zheng, Xi Chen, Glyn M. Rimmington

**Affiliations:** 1 Key Laboratory of Resource Plants, Beijing Botanical Garden, West China Subalpine Botanical Garden, Institute of Botany, Chinese Academy of Sciences, Xiangshan, Beijing, China; 2 University of Chinese Academy of Sciences, Yuquan Road, Beijing, China; 3 Key Laboratory of Agricultural Environment, Ministry of Agriculture, Institute of Environmental and Sustainable Development in Agriculture, Chinese Academy of Agricultural Sciences, South Zhongguancun Avenue, Beijing, China; 4 Xinjiang Institute of Ecology and Geography, Chinese Academy of Sciences, South Beijing Road, Urumqi, Xinjiang, China; 5 Global Learning College of Engineering, Wichita State University, Wichita Kansas, United States of America; DOE Pacific Northwest National Laboratory, United States of America

## Abstract

The variation of different ecosystems on the terrestrial carbon balance is predicted to be large. We investigated a typical arid region with widespread saline/alkaline soils, and evaluated soil respiration of different agricultural and natural ecosystems. Soil respiration for five ecosystems together with soil temperature, soil moisture, soil pH, soil electric conductivity and soil organic carbon content were investigated in the field. Comparing with the natural ecosystems, the mean seasonal soil respiration rates of the agricultural ecosystems were 96%–386% higher and agricultural ecosystems exhibited lower CO_2_ absorption by the saline/alkaline soil. Soil temperature and moisture together explained 48%, 86%, 84%, 54% and 54% of the seasonal variations of soil respiration in the five ecosystems, respectively. There was a significant negative relationship between soil respiration and soil electrical conductivity, but a weak correlation between soil respiration and soil pH or soil organic carbon content. Our results showed that soil CO_2_ emissions were significantly different among different agricultural and natural ecosystems, although we caution that this was an observational, not manipulative, study. Temperature at the soil surface and electric conductivity were the main driving factors of soil respiration across the five ecosystems. Care should be taken when converting native vegetation into cropland from the point of view of greenhouse gas emissions.

## Introduction

The dynamics of the amount of global carbon is the key issue of global warming, and soil carbon pools are correlated with carbon dioxide flux emission from soils [1]. Soil respiration is considered the largest terrestrial–atmospheric carbon exchange [2]. Any alterations in soil CO_2_ efflux could potentially exacerbate greenhouse-gas-induced climate warming [3]. Therefore, quantifying the seasonal and spatial variations in the CO_2_ efflux of different ecosystems is critical to understanding climate change. The temporal variations in soil respiration (Rs) can be characterized as diurnal/weekly, seasonal, annual, and centennial [4]. Because of the great variability on a temporal scale and the resulting measurement errors [5], it is necessary to measure Rs *in situ* in every month of growing season to accurately estimate annual Rs of different ecosystems.

Soil temperature, soil moisture and their interaction are regarded as the main controlling factors of Rs [3], [6], and they can explain most of the variation in Rs [7], [8]. Other factors such as alkalinity (pH) [9], salinity (electric conductivity, EC) [5], [10], and soil organic carbon (SOC) content [11] may affect how Rs interacts with temperature and moisture. Environmental factors such as temperature, moisture, sources and amounts of organic matter inputs will differ among different ecosystems [2], [12]. Therefore, Rs varies greatly with the ecosystem type. Furthermore, different amounts of litter production, litter quality, and root respiration under different vegetation regimes will affect soil respiration [13], [14].

The respiration rates for different ecosystems vary significantly, even between two adjacent plant ecosystems. Raich and Tufekcioglu found that under similar growing conditions, the soil respiration rate was 20% higher for grassland than that for forest [15]. Saviozzi et al. found similar results for adjacent crop fields, forests and grasslands having significantly different cumulative CO_2_ production from soil incubation [16]. Such findings indicate that different ecosystems must be considered in estimating the soil respiration rate of an area.

Arid regions occupy approximately 20% of the global terrestrial surface, and because of climate change, fire and ecosystem change, arid areas are expanding [17]. The amount of soil carbon stock in arid ecosystems is huge [1], and has been estimated to include 241 Pg of organic carbon and nearly the same amount or more inorganic carbon [18]. Because of the huge areas of arid regions and the large carbon pool they contain, carbon sequestration in arid regions is worthy of attention. However, few studies have estimated the carbon loss compared with other ecosystems in arid regions, and the lack of measurements in these regions adversely affects the estimation of global soil respiration.

The Xinjiang Uygar Autonomous Region (XUAR) in northwest China covers over one-sixth of China's land area and includes the majority of the country's arid areas [19]. Natural ecosystems were converted into agricultural ecosystems in huge area over the past 50 years, especially in the two basins of Taklimakan and Junggar [19]. Therefore Rs in the XUAR may have changed significantly, thus accelerating carbon cycle in China and even on a global scale. The Junggar basin is a typical “mountain–oasis–desert” system, and large amounts of salt on the soil surface are common owing to the salinity/alkalinity resulting mainly from land-use change. *Tamarix ramosissima* (Tr), *Haloxylon ammodendron* and *Reaumuria soongorica* (HR) are dominant shrub species in this region [5], while the non-native species *Phragmites communis* (Pc) is distributed over a large region of shallow underground water. To protect cropland and serve as a reforestation species for alkaline and sandy soils, *Elaeagnus angustifolia* (Ea) is widely cultivated as a farmland shelterbelt in the XUAR. Winter wheat (*Triticum aestivum*) (Wh) is one of the main grain crops grown in the XUAR, and is sowed in late September and harvested in August. The phenomenon of CO_2_ absorption in saline/alkaline soils reported by Xie et al. means that it is important to clarify Rs for different natural and artificial plant communities in the wide expanse of saline/alkaline soils in the XUAR [5].

The objective of this research was to investigate the effects of ecosystem types from natural communities to shelterbelt and crop ecosystems on Rs, and to clarify the relationships between Rs and soil temperature, moisture, and other environmental factors in a typical arid region. The study specifically addresses the question: What are the differences in diurnal and seasonal variations of Rs among different ecosystems? Particular attention is given to difference between natural ecosystems and agricultural ecosystems.

## Materials and Methods

### Ethics Statement

All necessary permits were obtained for the described field studies. The study sites are managed by the Fukang Station of Desert Ecology, Xinjiang Institute of Ecology and Geography, Chinese Academy of Sciences. The experiment has received the permits for all the field studies from the Fukang Station of Desert Ecology.

### Study area

The study was conducted in the Sangong River watershed, which is located at the southern edge of the Junggar basin. The study area has an arid climate with mean annual rainfall of 160 mm and mean annual temperature of 6.6°C [5]. The soils are very saline/alkaline.

The ecosystem is complicated in the oasis–desert area. According to the dominant natural vegetation and main artificial vegetation in the region, five ecosystems distributed in five adjacent sites were selected, including two artificial ecosystems (winter wheat cropland (Wh) and *E. angustifolia* shelterbelt (Ea)) and three natural ecosystems (*P. communis* grassland (Pc), *T. ramosissima* scrubland (Tr) and *H. ammodendron* + *R. soongorica* scrubland (HR)) (Table 1). Three randomly selected experimental plots (20 m×20 m) were established in each of the five ecosystems. The natural ecosystems were almost free from human disturbance.

**Table 1:** Descriptions of the study sites.

Ecosystems	Location	Management	pH	Main companion species composition
Winter Wheat (*Triticum aestivum*) crop land (Wh)	44°18' N87°51' E	Artificially management: irrigation several times with fertilizers	8.23±0.11	Few gramineous weeds
*Elaeagnus angustifolia* shelterbelt (Ea)	44°18' N87°51' E	Artificially management: irrigation two times annually without fertilizers	9.33±0.024	*Salsola collina*, *Karelinia caspica*, *Achnafherum splendens*, S*uaeda glauca*, *Limonium otolepis*
*Phragmites communis* grassland (Pc)	44°10'N 87°50' E	natural habit, no irrigation, adjacent to the cropland	9.41±0.19	*Halostachys caspica*, *Nitraria sibirica*
*Tamarix ramosissima* scrubland (Tr)	44°18' N87°52' E	natural habit, no irrigation	9.32±0.15	*Ceratocarpus arenarius*, *Salsola collina, Suaeda glauca*
*Haloxylon ammodendron* and *Reaumuria soongorica* scrubland (HR)	44°19' N87°50' E	natural habit, no irrigation	9.37±0.13	*Nitraria sibirica*, *Salsola collina*

### Soil CO_2_ flux measurements

Soil respiration was measured with an LI-8100 automated soil CO_2_ flux system equipped with an LI-COR 8100-103 chamber (LI-COR chamber volume of 4843 cm^–3^, Lincoln, Nebraska, USA). For better coverage of the spatial variability of soil respiration in the ecosystem, three plots in each experiment plot (20 m×20 m) were chosen for taking measurements. Taking the average distance between two dominant plants (*Da*) as a standard, Rs was measured (a) near the plant, (b) at 1/4 Da and (c) at 1/2 Da. Before setting up a PVC collar, litter on the soil surface was cleared and living vegetation inside the collar was clipped from the stem tip. In each ecosystem, nine PVC collars (three collars × three plots; 20.3 cm inner diameter and 15 cm high) were inserted into the soil with 3 cm exposed above the soil surface. Rs was measured at two hours interval from 8:00 to 20:00 at clear days every month during the growing season from May to October in 2010. As the winter wheat was harvested in August, the last measurements were made after the harvest at the Wh site. The 0-cm-depth (soil surface) and 10-cm-depth soil temperatures and 5-cm-depth volumetric soil water content (%) were measured automatically with a probe equipped with the LI-8100 system.

### Analysis of soil physical and chemical properties

Soil samples were collected at 0–30 cm soil depth. Three samples were obtained in each of the three plots, and then mixed up to three replicates according to the plot for the analysis. This work was performed synchronously with the Rs measurement in the five ecosystems. The soil samples were naturally dried and ground to pass through a 2-mm sieve. The pH (1∶5 solid–water ratio) and EC (1∶5 solid–water ratio) were then determined with a Eutech PC700 pH/EC meter (Thermo Fisher Scientific Inc., Waltham, Massachusetts, USA), and soil organic carbon (SOC) was measured using the methods described by Bao [20].

### Data analysis

The temperature dependence of Rs was fitted with Arrhenius and exponential functions:
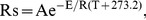
(1)where A is a fitted constant, E is the fitted apparent activation energy (J mol^–1^), R is the universal gas constant of 8.31 J mol^–1^ K^–1^, and T is the soil temperature (°C). The temperature coefficient Q_10_ was calculated as described [12]

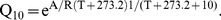
(2)For comparison, another widely used exponential model was used to fit the relationship between Rs and temperature [21]:
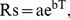
(3)where a and b are fitted constants, and T is the soil temperature (°C). Q10 was calculated using
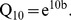
(4)Linear [7], quadratic [22] and exponential functions [23] were used to describe the relationship between Rs and soil moisture:



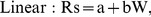
(5)

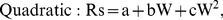
(6)

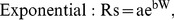
(7)where a, b and c are fitted constants, and W is the soil moisture at 5 cm depth.

An exponential-exponential function was used to describe effects of soil temperature and soil moisture on Rs [24], and model residuals were used to evaluate the model performance [25]:
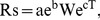
(8)where a, b and c are fitted constants, T is the soil temperature (°C), and W is the soil moisture.

All statistical linear and nonlinear regression analyses, multiple comparisons including one-way ANOVA and homogeneity of variance tests were performed with SPSS 13.0 software [26]. Multiple comparisons for means of different ecosystems were analyzed by Tukey test.

## Results

### Diurnal dynamics of the soil respiration rate

**Figure 1:**
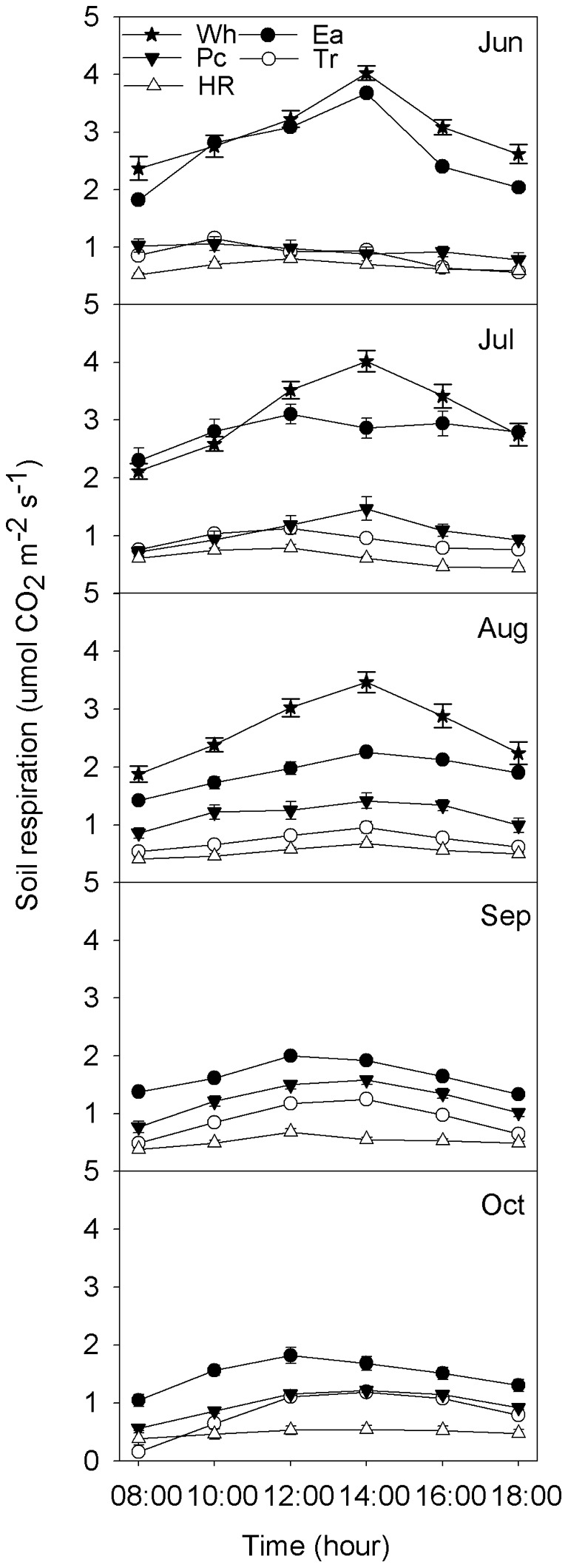
Diurnal dynamics of soil respiration rate (Rs) under different ecosystems. Wh =  Winter wheat (*Triticum aestivum*) crop land; Ea =  *Elaeagnus angustifolia* shelterbelt; Pc =  *Phragmites communis* grassland; Tr =  *Tamarix ramosissima* scrubland; HR =  *Haloxylon ammodendron* and *Reaumuria soongorica* scrubland. Data represent mean values ± SE from 9 repeats during the two-hour observed period.

The diurnal patterns of soil respiration in the five studied ecosystems were similar, having one-peak curves (Figure 1). The mean carbon dioxide flux in the nine repeated measurements of in every observation time was always positive, with fluxes being lowest in the early morning and highest at 12:00 or 14:00, which coincided with changes in the soil temperature at the surface (0 cm depth) better than changes in the temperature at 10 cm soil depth (Table 2). During the experiment periods, Rs for Wh, Ea, Pc, Tr and HR varied from 4.03±0.13 to 1.89±0.07, 1.04±0.10 to 3.67±0.08, 0.56±0.04 to 1.58±0.07, 0.16±0.04 to 1.25±0.04 and 0.38±0.01 to 0.80±0.06 µmol CO_2_ m^–2^ s^–1^, respectively. Wh had the highest Rs, while HR had the lowest Rs. When the soil temperature decreased to 5°C at 8:00 in October, Rs for some plots became negative (0 to –0.3 µmol CO_2_ m^–2^s^–1^, mean value for one time observation was positive) for Tr, Pc and HR but not for Ea and Wh.

**Table 2:** Fitted parameters of exponential and Arrhenius functions for the relationship between Rs (µmol CO_2_ m^–2^ s^–1^) of Wh, Ea, Pc, Tr, HR, and temperature (T) at soil surface.

Site codes	Functions	R^2^	*P*	Q_10_
Wh	Rs = 1.36e^0.019T^	0.508	**	1.21
	Rs = 989e^−15176/R(T+273.2)^	0.514	**	1.20
Ea	Rs = 0.884e^0.0304T^	0.680	**	1.36
	Rs = 13242e^−21935/R(T+273.2)^	0.678	**	1.34
Pc	Rs = 0.412e^0.0307T^	0.809	**	1.36
	Rs = 9253e^−22931/R(T+273.2)^	0.560	**	1.12
Tr	Rs = 0.303e^0.0287T^	0.528	**	1.33
	Rs = 6652e^−23018/R(T+273.2)^	0.560	**	1.33
HR	Rs = 0.330e^0.0142T^	0.483	*	1.15
	Rs = 46.7e^−11406/R(T+273.2)^	0.490	*	1.14

Temperature for Wh, Ea, Pc, Tr and HR ranged from 22.0 to 51.9, 3.5 to 46.9, 9.7 to 54.2, 9.5 to 52.5 and 9.5 to 53.9°C, respectively.

* means *p*<0.05, ** means *p*<0.01. All data were shown in Fig. 1. See table 1 for site code definitions.

### Seasonal dynamics of Rs

**Figure 2:**
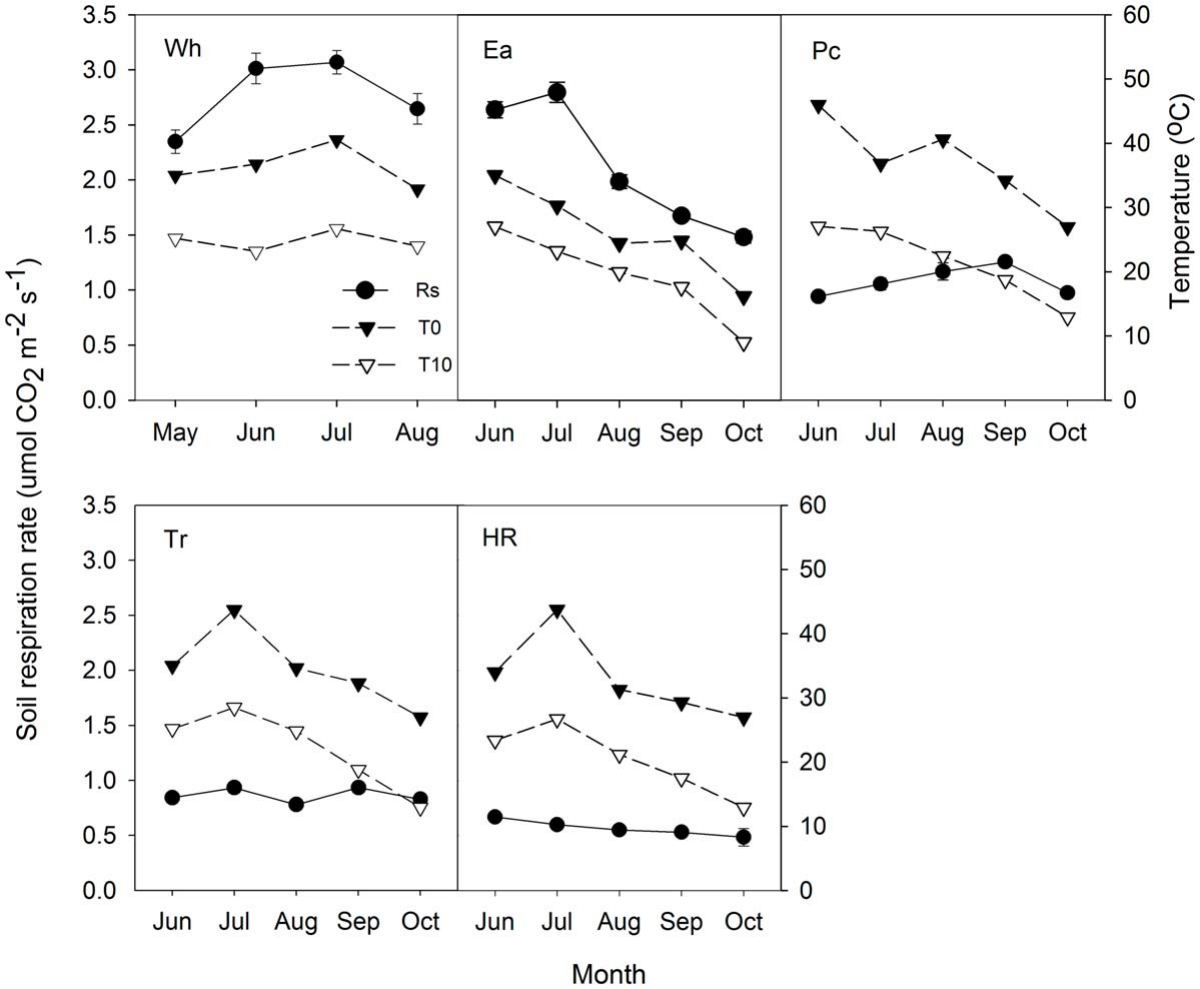
Seasonal dynamics of Rs and soil temperature for Wh, Ea, Pc, Tr and HR. T0 =  soil temperature at soil surface; T10 =  soil temperature at 10 cm depth. Rs data represent mean ± SE on the measured day (n = 6). Other abbreviations are same as Figure. 1.

Rs for Wh, Ea, Tr and HR had a single-peak curve, but that for Pc had a minimum in August owing to the low soil water content (11%) (Figure 2).

Rs for Wh, Ea and HR peaked in July and then decreased with temperature. In contrast, Rs for Pc peaked in September and was lowest in October.

**Figure 3:**
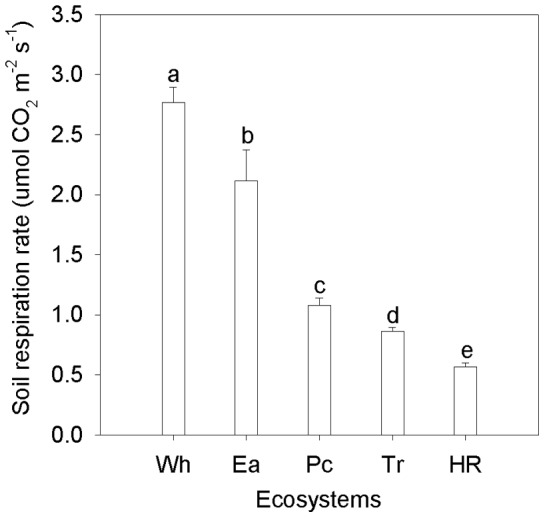
Rs (mean ± SE) in the whole growing season for five ecosystems. Each bar represents the mean of six replicates; bars with different lowercase letters are significantly different from each other with different ecosystems at *p*<0.05 (Tukey Test). Other abbreviations are same as Figure. 1.

The average Rs values in the growing season were 2.77, 2.11, 1.07, 0.86 and 0.57 µmol CO_2_ m^–2^ s^–1^ for Wh, Ea, Pc, Tr and HR, respectively. One-way ANOVA showed that there were significant differences among Rs values of the five ecosystems (*P*<0.05) (Figure 3). Rs for Ea had greater seasonal variation than values for the other four ecosystems.

### Relationship between Rs and temperature

For the five ecosystems, the best fitted relationships were obtained using soil temperature at 0 cm depth, which explained 48.3% to 80.9% and 49.0% to 67.8% of the variation in Rs when using exponential and Arrhenius functions, respectively. For the relationship between Rs and temperature at 10 cm depth, the regression was effective (*P*<0.05) only for the Ea ecosystem when using either of the two models. The regressions explained 49.8% and 49.0% of Rs variation, which were lower than the values obtained using the temperature at 0 cm.

Q_10_ for the five ecosystems ranged from 1.15 to 1.36 when using the exponential model. Q_10_ calculated with the Arrhenius function was similar, ranging from 1.14 to 1.34. The highest and lowest values of Q_10_ were for Ea and HR, respectively.

### Relationship among soil respiration and soil moisture and soil temperature

When soil moisture was taken as a single controlling factor of Rs, linear, quadratic and exponential equations did not explain the Rs variation for Wh, Pc, Tr and HR ecosystems. However, for Ea, the soil moisture explained more than 46% of the Rs variation (*P*<0.01) when using the three equations.

**Figure 4:**
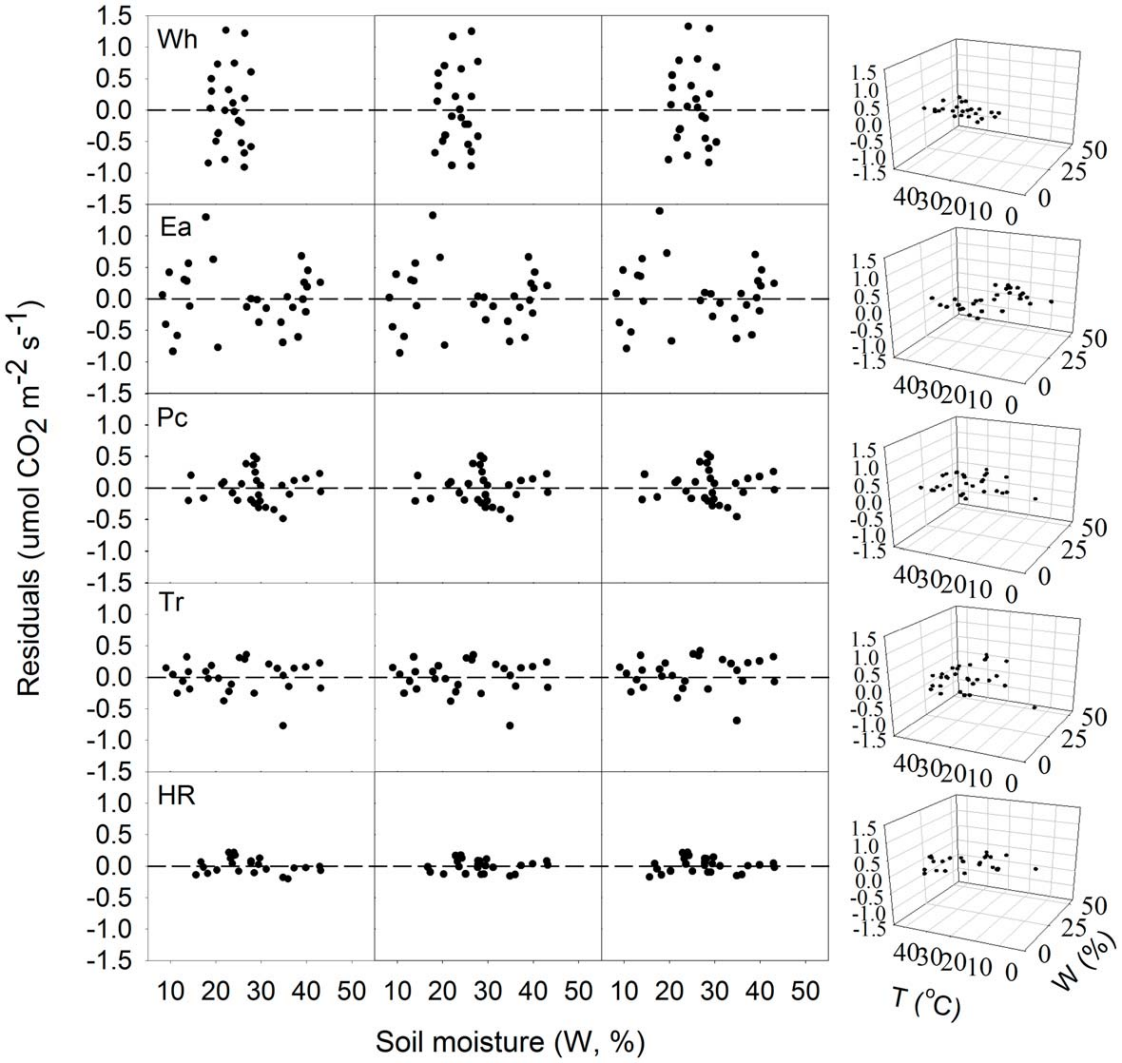
Residuals from soil moisture equations vs. soil moisture or/and temperature. Panels within a row refer to the same site; panels within a column refer to the same equation (equations 5–8, respectively). The soil temperature was at soil surface and soil moisture was at 5 cm soil depth. Other abbreviations are same as Figure. 1.

Temperature at 0 cm and soil moisture together could improve the correlation coefficients of the regression equation for Rs (*R* ^2^ = 0.27–0.86) in the five ecosystems (Table 3). The residual distributions of these models also indicated a well simulating of Rs with both soil temperature and soil moisture (Figure 4).

**Table 3:** Fitted relationships of soil respiration (µmol CO_2_ m^–2^ s^–1^) with soil moisture (at 5 cm soil depth) (W, %) and soil temperature (at soil surface).

Site codes	Functions	R^2^	*P*
Wh	Linear: Rs = 2.54+0.01W	0.023	0.82
	Quadratic: Rs = −3.73+0.56W-0.12W^2^	0.023	0.78
	Exponential: Rs = 2.54e^0.03W^	0.001	0.86
	Exponential – Exponential: Rs = 1.09e^0.009W^ e^0.019T^	0.52	**
Ea	Linear: Rs = 3.05-0.038W	0.47	**
	Quadratic: Rs = 3.2-0.053W+3.05E^−04^W^2^	0.47	**
	Exponential: Rs = 3.16e^−0.018W^	0.46	**
	Exponential – Exponential: Rs = 1.28e^−0.09W^ e^0.025T^	0.86	**
Pc	Linear: Rs = 1.31-0.008W	0.05	0.24
	Quadratic: Rs = 1.365-0.011W+4.4E^−05^W^2^	0.05	0.51
	Exponential: Rs = 1.34e^−0.008W^	0.055	0.22
	Exponential – Exponential: Rs = 0.77e^−0.003W^ e^0.012T^	0.27	*
Tr	Linear: Rs = 0.764+0.005W	0.045	0.27
	Quadratic: Rs = 0.70-6E^−05^W+0.010W^2^	0.045	0.55
	Exponential: Rs = 0.78e^0.002W^	0.005	0.72
	Exponential – Exponential: Rs = 0.28e^0.001W^ e^0.031T^	0.57	**
HR	Linear: Rs = 0.61-0.001W	0.015	0.56
	Quadratic: Rs = 0.715−0.001W+−3.78E^−04^W^2^	0.25	0.051
	Exponential: Rs = 0.702e^−0.008W^	0.088	0.14
	Exponential – Exponential: Rs = 1.09e^0.009W^ e^0.019T^	0.61	**

* means *p*<0.05, ** means *p*<0.01. See table 1 for site code definitions.

### Variation in soil respiration related to EC and SOC content

**Figure 5:**
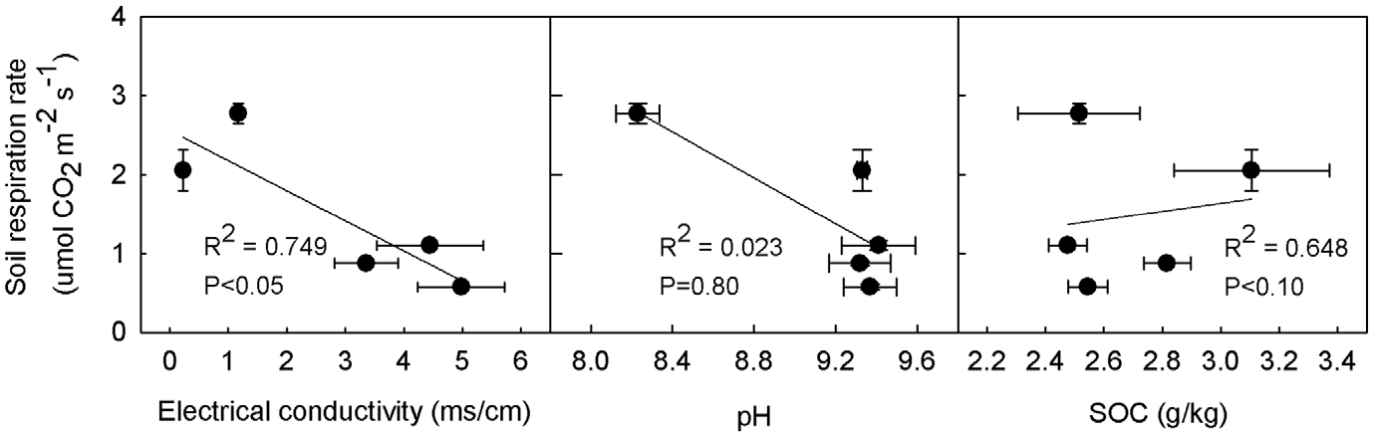
Relationship between Rs and EC, pH, SOC at 0-30cm soil depth. EC =  electric conductivity; SOC =  soil organic carbon contents. Rs data represent mean ± SE in the whole growing season (n = 9). EC, pH, SOC data represent mean ± SE (n = 3). Each point represent individual site including Wh, Ea, Pc, Tr and HR.

Ea had the highest SOC content and lowest EC (Figure 5). Rs had a significantly negative correlation with EC (*P*<0.05), and the variation in Rs among the five ecosystems could be explained largely by EC (*R* ^2^ = 0.749). However, the correlation between Rs and SOC content was not significant (*P* = 0.80).

## Discussion

### Variation of Rs in different ecosystems

Various ecosystems are known to affect the terrestrial carbon dynamics in different ways [27]. Raich and Schlesinger obtained mean values of annual soil respiration for different ecosystems: 442 g°C m^−2^ yr^−1^ for temperate grasslands, 224 g°C m^−2^ yr^−1^ for desert scrub and only 60 g°C m^−2^ yr^−1^ for tundra [2]. In comparisons of grasslands and forests growing under the same conditions, Raich and Tufekcioglu found that the grasslands had Rs 20% higher than those of comparable forest stands [15]. Similar to the results reported in previous studies, our investigation of the five ecosystems showed significant differences in Rs, although we caution that this was an observational, not manipulative, study. Furthermore, the five sites selected in the experiment were adjacent and agricultural ecosystems were originally similar to adjacent natural ecosystems, suggesting that the variations in Rs might be due to ecosystem changes and not other factors correlated with spatial variations. Similar results have been reported for side-by-side comparisons of different plant ecosystems [15].

The mean Rs values for the whole growing season in the two natural ecosystems Tr and HR dominated by native species were 0.86 and 0.57 µmol CO_2_ m^–2^ s^–1^, respectively. These values were close to the soil respiration result of 0.58 µmol CO_2_ m^–2^ s^–1^ obtained for desert ecosystems in northwest China [28]. However, in an ecosystem dominated by the non-native species *Phragmites communis*, the mean Rs was 1.08 µmol CO_2_ m^–2^ s^–1^, which is significantly higher than the rates for Tr and HR in this study. This might relate to the survival strategies of the species. *P. communis* has phalanx clonal growth morphology and can grow dense roots, rhizomes and aboveground tillers [29], possibly leading to more root biomass in the shallow soil layer and a higher mean soil respiration rate for Pc than for Tr and HR.

Cultivation activities by humans have greatly affected global carbon sequestration [2]. With the conversion of natural landscapes of ecosystems under artificial management, Rs has been found to increase in agroecosystems and shelterbelts [30], [31], [32]. Compared with Rs values for natural ecosystems, the mean soil respiration for Wh and Ea was 159%–386% and 96%–273% higher, respectively. To quickly obtain more resources such as food, fiber and other economic resources, natural ecosystems were converted into agricultural ecosystems in the XUAR. These practices might increase Rs and greatly affect the terrestrial carbon balance [15]. There have already been obvious changes in ecosystems, and future changes are likely to occur. Therefore, proper management and conservation measures should be considered to prevent the reduction of soil carbon.

Xie et al. reported that alkaline/saline soils can absorb CO_2_ under natural conditions [5]. In our experiment, we recorded negative flux only when the temperature was below 5°C in October in the three natural ecosystems, and the flux (0 to –0.3 µmol CO_2_ m^–2^ s^–1^) was similar to that obtained by Xie et al. at a similar time [5]. Regarding negative soil respiration, Xie et al. confirmed that CO_2_ is absorbed by saline/alkaline soil [5], and others considered that it is correlated with lower air temperature [33]. Further work focusing on this phenomenon will be valuable because of the spread of saline/alkaline soil in the XUAR, and it may also have implications for global carbon sequestration. Furthermore, the negative CO_2_ flux of soil only being observed in natural ecosystems in this experiment might imply that the change from natural ecosystems to agricultural ecosystems will decrease CO_2_ absorption by soil, and this phenomenon should be noted in addressing global warming and cropland exploration in the XUAR.

### Effect of temperature and moisture on soil respiration

Soil temperature and moisture are usually taken as important factors in controlling soil respiration and they can indeed explain most of the variation in soil respiration [8], [12].

Temperature can affect almost every aspect of soil respiration [4]. However, the choice of soil depth at which temperature is measured has to be considered. The temperature at 10 cm depth has been used to describe relationships between temperature and soil respiration by many researchers [31], [34]. Owing to seasonal and diurnal fluctuations in radiation and air temperature, the temperature fluctuations are greatest at the soil surface [35]. Meanwhile, temperature fluctuations at increasing soil depth increasingly lagged behind temperature fluctuations at the surface [36]. Therefore, the choice of a suitable soil depth to measure temperature for predicting soil respiration is a significant challenge. In our study, we found that soil respiration in all five ecosystems correlated better with the soil surface temperature than with the 10-cm-depth temperature. When regressing the soil respiration and the 10-cm-depth temperature with the exponential and Arrhenius functions, the correlations were found to be significant only for Ea. Using the optimized coefficients of regression for soil respiration and soil surface temperature (*R* ^2^: 0.48–0.81), the surface temperature might be an appropriate indicator of the fluctuations of soil respiration in the study area. Similarly, Pavelka et al. reported that the soil surface was the most suitable depth for recording the soil temperature because it has the best coefficient of correlation with soil respiration and temperature [36].

Q_10_ is commonly used to describe the sensitivity of soil respiration to temperature among different sites [36]. Depending on the location and ecosystem type, values of Q_10_ vary widely [21]. Bond-Lamberty and Thomson suggested a global moderate Q_10_ value of 1.5 [37], and Mahecha et al. reported that it is around 1.4 [38]. In the five ecosystems studied in the arid area, Q_10_ varied from 1.15 to 1.36 as calculated with the exponential function and from 1.14 to 1.34 as calculated with the Arrhenius function, which suggests that Rs generally increases with temperature; however Q_10_ has lower sensitivity than the global Q_10_ mentioned above. Because of the inadequacy of the exponential function in the regression of temperature and soil respiration for Pc, we used the Arrhenius function. Owing to the high level of water–salt stress in a natural habitat, the soil microbial communities, biomass and efficiency were affected by the ecosystem type [31], [39], and these variations could result in lower temperature sensitivity for soil respiration. The hypothesis of Thornley and Cannell states that stable carbon has less temperature sensitivity than labile carbon [39]. If this was tested, it could further explain the higher temperature sensitivity of soil respiration for cultivated lands that have more labile carbon in soil owing to till management.

Soil moisture is another widely accepted factor affecting Rs at the ecosystem level [6]. Bowden et al. found that Rs is only limited by extremes of soil moisture [40]. Liu et al. assume that there is an optimal plateau of the soil moisture content for Rs that represents a range near the intermediate soil moisture level [41]. In the areas investigated in this study, the soil moisture content had a wide range from 8% to 43% and no significant optimal moisture levels were found. Regressions using linear, quadratic and exponential models for Rs and soil moisture content indicated that, as a single factor, soil moisture failed to explain the variations in Rs in the growing season for the three natural ecosystems. For the shelterbelt ecosystem, the relationship between Rs and moisture content was well modelled by linear, quadratic and exponential functions (*P*<0.01). Not considering other environmental factors, the soil moisture content correlated negatively with Rs; and when the soil moisture content exceeded 20%, Rs became relatively steady. This phenomenon illustrated that moisture content of 20% might be the threshold for the Ea ecosystem, similar to the findings of Rey et al. [34]; Rs flux increases with moisture content below this threshold, and remains steady above the threshold. However, for the cultivated Wh ecosystem, there was almost no correlation between Rs and soil moisture. We speculate that this was due to the more careful management of the agroecosystem, for which there was no water stress.

Because soil moisture only slightly explained variations in Rs, we added temperature and the interactions of temperature with the soil moisture to the Rs–soil moisture exponential-exponential model. It was found that this approach better predicts variations in Rs for all five ecosystems. Similar results have been found in many earlier works [7], [8]. Comparing the models used in the analysis, we find that Rs is well related to surface temperature at any particular soil moisture content except for Wh, because of the higher explanation percentages for the surface temperature. Similarly, Luo and Zhou reported that when there is ample substrate in the growing season, Rs is more affected by temperature [4].

### Effects of EC, pH and SOC content on soil respiration

As soil respiration is a complex process, many environmental factors including salinity (EC), alkalinity (pH) and nutrient supply could affect Rs. Salinity is a severe problem in arid and semiarid regions, and is usually accompanied with high alkalinity, because of calcium carbonate or hydrolysis of sodium carbonate enrichment in the uppermost soil layers [42]. Salinity/alkalinity can affect Rs through strong effects on the microorganisms including effects on microbial biomass, population, community structure and activity [42], [43]. However, contradictory results have been obtained for the effects of salinity/alkalinity on soil microbial activity (respiration rate), including depressive effects [10]; stimulation with increasing sodicity and inhibition with increasing salinity [44]; and stimulation at low salt concentrations and inhibition at higher stress [45].

In this study, the EC values significantly decreased for Wh and Ea, mainly because of irrigation management that shifted salt into the drainage channel. There was significant negative correlation between mean Rs in the whole growing season and EC, and it can explain 74.9% of the variation among the five ecosystems for the linear function. This may be attributed to the depressive effects on the microorganisms mentioned above. Similar results for the inhibition effects of salinity on RS have been obtained in many previous works [5], [10], [42].

pH in the saline/alkaline soils did not explain Rs variations among ecosystems as well as EC did. Although the regression for the mean Rs in the whole growing season and pH was only significant at the 0.10 level, it may indicate that alkalinity was a depressive factor of Rs. Similar results have been reported that Rs correlates negatively with pH when the pH value exceeds 7.0 [9].

In theory, SOC content is another important factor affecting Rs because it can supply substrates to microbial heterotrophs [11]. Brooks et al. found that Rs positively correlates with the SOC content [46]. However, the linear regression for Rs and SOC content indicates that the SOC content fails to explain variations in Rs among ecosystems (Figure 5). Additionally, we found that for agricultural ecosystems compared with natural ecosystems Rs but not the SOC content significantly increased. This may explained the lower correlation between Rs and SOC content, and might indicate that the SOC content was not the determinant factor of variations in Rs among natural and agricultural ecosystems.

### Conclusions

Seasonal variations in Rs were well explained by the soil surface temperature but not by the 10-cm depth soil temperature or soil moisture, and the Rs sensitivity to temperature (Q_10_) was lower in agricultural ecosystems compared with natural ecosystems. In contrast to the case for many other ecosystems, the SOC content failed to explain variations in Rs among ecosystems. However, EC explained Rs variations among ecosystems, and this has implications for future work, especially studies on the wide spread of saline/alkaline areas in the XUAR. Given the size of the XUAR and other similar arid regions around the world, small changes in soil respiration following conversion of natural ecosystems would have remarkable implications for the regional emissions of CO_2_ flux to the atmosphere. Proper measurements will be needed to better manage the balance between immediate human needs and maintaining carbon sequestration.
